# Interventions for adolescent depression comorbid with non-suicidal self-injury: a scoping review

**DOI:** 10.3389/fpsyt.2025.1601073

**Published:** 2025-06-26

**Authors:** Shihan Fang, Fazhan Chen, Jing Bian, Lei Zhang, Yanbo Wang

**Affiliations:** ^1^ School of Medicine, Tongji University, Shanghai, China; ^2^ Affiliated Mental Health Center & Hangzhou Seventh People’s Hospital, Zhejiang University School of Medicine, Hangzhou, China; ^3^ Clinical Research Center for Mental Disorders, Shanghai Pudong New Area Mental Health Center, School of Medicine, Tongji University, Shanghai, China

**Keywords:** adolescent depression, non-suicidal self-injury, scoping review, dialectical behavior therapy, family-system intervention, combined therapy, emotion regulation, neuroinflammatory pathways

## Abstract

**Background:**

Adolescent depression and non-suicidal self-injury (NSSI) represent significant global public health challenges, with high comorbidity rates and multidimensional adverse outcomes. Despite growing evidence on interventions, a comprehensive synthesis of strategies targeting this co-occurrence remains limited.

**Aim:**

This scoping review systematically maps evidence-based interventions for adolescents with comorbid depression and NSSI, focusing on efficacy, mechanisms, and implementation challenges.

**Methods:**

Following Arksey and O’Malley’s framework and PRISMA-ScR guidelines, a systematic search of English and Chinese databases (2000–2025) identified 18 studies (13 randomized controlled trials [RCTs], 3 quasi-experimental studies, and 2 other study designs). Data were extracted and synthesized to characterize intervention types, outcomes, and contextual factors.

**Results:**

Psychotherapies, particularly dialectical behavior therapy for Adolescents (DBT-A), demonstrated robust efficacy, reducing NSSI frequency by 50% and relapse rates through enhanced emotion regulation. Family-system approaches (e.g., Satir therapy) improved family cohesion and reduced comorbid behaviors. Pharmacological agents (e.g., Selective Serotonin Reuptake Inhibitors) showed synergistic effects when combined with psychotherapy, while neuromodulation (e.g., Repetitive Transcranial Magnetic Stimulation) normalized neuroinflammatory markers. Innovations like narrative therapy facilitated identity reconstruction by externalizing NSSI as a separate entity from self-concept. Key challenges included cultural adaptability, limited long-term follow-up (≥1 year), and understudied digital intervention roles.

**Conclusions:**

Integrated biological-behavioral interventions, culturally tailored protocols, and family-system strategies are pivotal for managing NSSI-depression comorbidity. Future research should prioritize rigorous RCTs with extended follow-up periods, community-based implementation, and digital mental health solutions to address scalability and sustainability gaps.

## 1 Introduction

Depressive disorders and non-suicidal self-injury (NSSI) among adolescents have emerged as pressing global public health challenges in recent decades. Major depressive disorder, characterized by persistent low mood, anhedonia, and reduced volitional activity, demonstrates particular clinical significance during the critical developmental transition of adolescence (1). According to the World Health Organization (WHO) – encompassing 194 member states across all regions – depressive disorders constitute the leading cause of disability-adjusted life years (DALYs) in adolescents worldwide, with current prevalence estimates ranging from 10% to 20% in community samples (2). A meta-analysis synthesizing 72 studies further substantiates these concerns, reporting a global point prevalence of 34% for clinically significant self-reported depressive symptoms in adolescent populations, with epidemiological trends indicating progressive escalation (3). Notably, female adolescents and those residing in Middle Eastern, African, and Asian regions demonstrate particularly elevated prevalence rates (3, 4). The clinical trajectory of adolescent depression is characterized by multidimensional impairment, including psychological distress, social dysfunction, academic decline, and reduced quality of life. Critically, this condition significantly increases suicide risk, causing substantial individual suffering and imposing considerable socioeconomic burdens on families and society (5).

Non-suicidal self-injury (NSSI) is defined as deliberate self-inflicted physical harm without suicidal intent, typically manifested through cutting, burning, or self-impact behaviors (6). Recognized as a critical clinical concern, NSSI has been included in the ‘Conditions for Further Study’ section of the Diagnostic and Statistical Manual of Mental Disorders, Fifth Edition (DSM-5), accompanied by provisional diagnostic criteria to facilitate systematic investigation (7). Epidemiological evidence highlights the elevated prevalence of NSSI among adolescents globally, with a lifetime prevalence of 19.3% (8)and 15-20% of adolescents reporting at least one self-injury episode (9). Notably, Chinese adolescents exhibit an even higher prevalence rate of 27.4% (10). Emerging data reveal significant psychiatric comorbidity patterns, particularly bidirectional associations between NSSI and depressive symptoms. A nationwide Chinese study involving 8,102 adolescents demonstrated that the NSSI group had a markedly higher depression detection rate (49.7%) compared to non-NSSI counterparts (17.7%), with females and high school students constituting vulnerable subgroups (11). Strikingly, 40% of adolescents diagnosed with major depressive disorder engage in NSSI behaviors (12), substantially exceeding population baselines.

Critically, NSSI functions not merely as a behavioral marker but as an active contributor to psychopathological progression. A multi-wave prospective study confirms that NSSI exacerbates depressive symptomatology through maladaptive feedback loops while serving as a robust predictor of subsequent suicide attempts (13). Furthermore, NSSI demonstrates strong comorbidity with anxiety disorders, post-traumatic stress disorder (PTSD), and borderline personality disorder (14, 15), collectively amplifying its burden across multiple mental health domains. These findings underscore the dual disease burden arising from the NSSI-depression syndemic and emphasize the imperative for targeted clinical interventions.

Existing interventions exhibit substantial heterogeneity (16–18). Dialectical Behavior Therapy for Adolescents (DBT-A) has demonstrated robust efficacy in reducing NSSI recurrence through emotion regulation skills (19, 20). Internet-based interventions show promise in overcoming geographical barriers, though their effectiveness in severe cases remains understudied and limited by digital divides (21, 22). A prospective randomized controlled trial revealed that individual narrative therapy maintained significant reductions in NSSI behaviors at 1-month follow-up, though therapeutic effects were moderated by familial support levels and patients’ cognitive flexibility (23). Pharmacological interventions, particularly selective serotonin reuptake inhibitors (SSRIs) and second-generation antipsychotics (SGAs), show limited target-specific evidence for NSSI reduction despite their antidepressant properties, with additional concerns regarding treatment response fluctuations and adverse effect profiles (24, 25).

Cross-cultural analyses further indicate that racial disparities in depression attribution models and treatment preferences may substantially influence intervention adherence. For instance, African American families demonstrate greater receptivity to cognitive behavioral approaches than biomedical explanations, underscoring the necessity for culturally adaptive modifications in standardized protocols (26). Notably, the social contagion effects of digital platforms on NSSI propagation remain inadequately addressed, with emerging evidence suggesting online communities may normalize self-injurious behaviors (27). Although prior systematic reviews have extensively investigated interventions for suicidal behavior (28), a significant gap persists in evidence-based strategies specifically targeting depression with NSSI behavior. Therefore, our scoping review systematically maps evidence-based interventions for adolescent depression with comorbid NSSI, employing rigorous methodology to characterize outcome measures, treatment efficacy, and implementation challenges through an interdisciplinary lens.

## 2 Methods

### 2.1 Aim

The aim of this scoping review is to systematically map evidence-based interventions for adolescent depression with comorbid non-suicidal self-injury (NSSI) and address three key research questions:

- What interventions demonstrate efficacy in reducing depressive symptoms and NSSI behaviors?
- How do contextual factors (e.g., cultural adaptability, interdisciplinary collaboration) influence intervention effectiveness?
- What gaps persist in long-term outcomes, scalability, and digital health integration?

This synthesis seeks to inform translational research and optimize precision intervention frameworks through rigorous characterization of outcome measures, efficacy evidence, and implementation challenges.

### 2.2 Design

A scoping review is a comprehensive method following evidence-based practice principles. This methodology was selected as it allows for systematic mapping of emerging evidence in adolescent mental health interventions, particularly given the heterogeneous nature of existing studies in terms of design, population, and intervention types. Scoping reviews are uniquely suited to identify research gaps and clarify conceptual boundaries in complex, multidisciplinary fields—a critical need in this domain. It quickly helps researchers grasp the research status in a specific field, clarify the sources and types of existing evidence, summarize and analyze relevant outcomes, and identify research gaps (29). This scoping review adhered to (30) methodological framework, which comprises four core phases: a) systematically identifying relevant studies through database searches, b) applying predefined inclusion criteria for study selection, c)organizing extracted data in a structured format, and d) synthesizing findings via comprehensive thematic analysis. To ensure methodological rigor and reporting standardization, the investigation strictly adhered to the PRISMA-ScR guidelines (31), a recognized reporting framework specifically designed for scoping reviews. To enhance methodological transparency, we incorporated a brief critical appraisal of study heterogeneity (e.g., variability in sample sizes, intervention durations, and outcome measurement tools) and explicitly acknowledged limitations related to risk of bias across studies (e.g., lack of blinding in quasi-experimental designs). Given the nature of this secondary analysis which exclusively involved synthesizing published research findings, ethical committee approval was formally waived throughout the review process. The protocol for this scoping review is registered on the Open Science Framework (OSF; https://osf.io/kdb7x) with DOI: https://doi.org/10.17605/OSF.IO/QTFMZ.

### 2.3 Search strategy

Our search strategy spanned both English and Chinese scholarly databases to ensure comprehensive coverage of research on adolescent mental health interventions. Specifically, we queried English databases—PubMed, PsycINFO, CINAHL, Web of Science, and Scopus—as well as Chinese repositories such as CNKI and Wanfang. Moreover, by restricting our search to publications from January 2000 to January 2025, we captured a critical period marked by significant advances and evolving paradigms in adolescent mental health intervention strategies.

A search was conducted using both subject headings and free-text terms, and the references of the included studies were tracked. The subject headings comprised ‘adolescents’ ‘depression’ ‘non-suicidal self-injury’ and ‘intervention’. Keywords were selected by integrating MeSH terms, free-text terms, truncation, and synonyms. Boolean operators (AND, OR, NOT) were employed to construct the search strategy, ensuring both its comprehensiveness and accuracy. The detailed search strategy and the final search strategy for Web of Science are shown in **Table 1**. The search strategies for other databases are available from the authors on request.

**Table 1:** Search strategy.

Subject term	Key words or Synonyms or Extended Word
Adolescent(=a)	"adolescent" OR "Adolescen*" OR "teenager" OR "youth" OR "juveniles" OR "teen" OR "middle school student"
Depression(=b)	"depression" OR "depressive disorder" OR "depressed" OR "depress*" OR "MDD"
Non-Suicidal Self-Injury(=c)	"non-suicidal self-injur*" OR "NSSI" OR "deliberate self-harm" OR "self-injurious behavior" OR "intentional self injury" OR "intentional self harm" OR "self-destructive behavior" OR "self-harm" OR "self-injury"
intervention (=d)	"interven*" OR "intervention strategies" OR "psychological interventions" OR "behavioral interventions" OR "program*" OR "therap*"OR "emotion regulation strategies" OR "prevention measures" OR "symptom management" OR "self-management" OR "behavioral control" OR "symptom control" OR "intervention evaluation" OR "risk assessment" OR " clinical measures" OR "Methods" OR"healthcare strategies"
Search Logic	"a" AND "b" AND "c" AND "d"
Databases	PubMed, Web of Science, Scopus, PsycINFO, Cochrane Library, CNKI,Wanfang
Search Field	title, abstract
Conducted Time	January-2000~ February-2025
For example (pubmed)	((("adolescent"[Mesh] OR "teenager" OR "youth" OR "juveniles" OR "teen" OR "middle school student" OR "Adolescen*") AND ("depression"[Mesh] OR "depressive disorder"[Mesh] OR "depressed" OR "depress*" OR "MDD")) AND ("non-suicidal self-injur*" OR "NSSI" OR "deliberate self-harm" OR "self-injurious behavior"[Mesh] OR "intentional self injury" OR "intentional self harm" OR "self-destructive behavior" OR "NSSI" OR "self-harm" OR "self-injury")) AND ("interven*" OR "intervention strategies" OR "psychological interventions" OR "behavioral interventions" OR "program*" OR "therap*"OR "emotion regulation strategies" OR "prevention measures" OR "symptom management" OR "self-management" OR "behavioral control" OR "symptom control" OR "intervention evaluation" OR "risk assessment" OR " measures" OR "Methods"[Mesh] OR “healthcare strategies”)

### 2.4 Study selection

Study screening was conducted in two steps: 1) title and abstract screening and 2) full text screening. Prior to each step, the Program Manager (YBW) trains researchers to pilot test a sample of 30 studies to ensure consistency in screening. Thereafter, the project manager (FZC) rigorously monitors the screening process and provides feedback to the researchers to further improve the accuracy of the screening.

The inclusion and exclusion criteria for screening the literature are detailed in **Table 2**. It is important to note that a clear description of the intervention content and implementation process means that selected papers need to include specific measures and outcome indicators. If the description of the intervention is incomplete (e.g., lack of specific implementation steps or failure to report outcome indicators), the literature should be excluded. Regarding the classification of adolescent age, given the World Health Organization’s definition of adolescence as ages 10 to 19 (32), any study whose subjects fall outside this age range should be excluded. Non-peer-reviewed grey literature (for example, conference abstracts, research protocols, guidelines, opinions, case studies, policy documents) was excluded. It is methodologically justifiable to permit the inclusion of studies without strict differentiation between suicide and NSSI, particularly when the research objectives have limited relevance to the necessity of distinguishing these behavioral manifestations.

**Table 2:** Inclusion and exclusion criteria.

Inclusion criteria	Exclusion criteria
Study subjects were adolescents with depression and/or NSSI	Inaccessible full texts
Study design including randomized controlled trials, quasi-experimental studies, mixed method	Reviews, systematic evaluations,
Strategies or Intervention content was clearly defined	Assessment of outcome measures for absence of self-injury
	Without strict suicide/NSSI differentiation (notably when distinction is critical to research aims)
	Publications not in English or Chinese languages
	Duplicate publications
	Qualitative study
	Non-peer-reviewed grey literature (e.g., conference abstracts, research protocols, guidelines, comment, policy documents)

**Figure 1:**
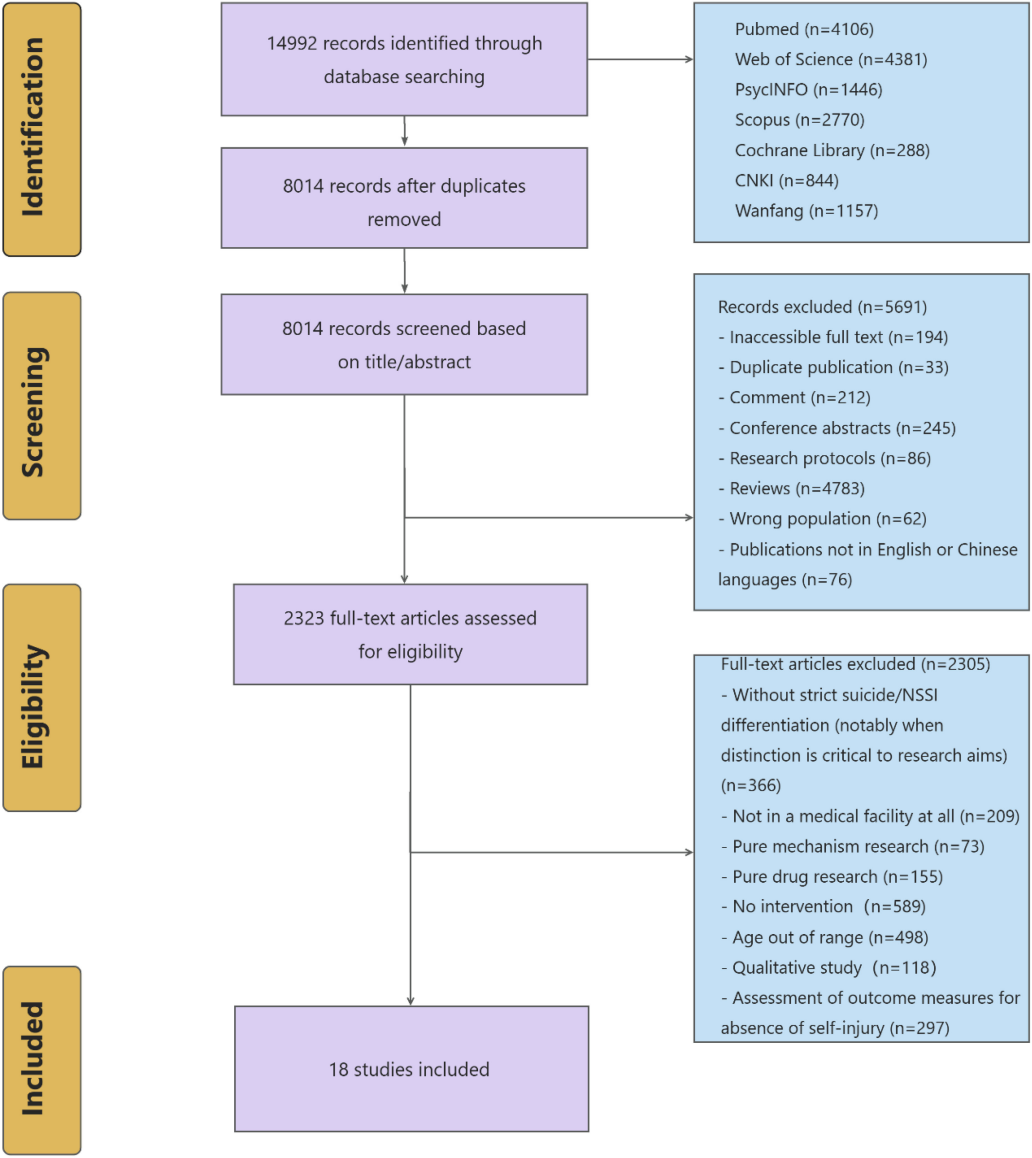
PRISMA flowchart of intervention of adolescent depression patients with non-suicidal self-injury behavior.

Firstly, the retrieved literature was imported into Covidence to remove duplicates. After deduplication, the initial screening of titles and abstracts was conducted. Following this initial screening, a second round of full-text screening was performed to confirm eligibility. The preliminary screening of titles and abstracts was systematically executed by four principal investigators (SHF, JB, LZ, YBW) employing a dual-reviewer verification protocol. Utilizing a paired evaluation framework, each dyad operated through coordinated workflows: the primary reviewer conducted initial eligibility assessments while the secondary researcher performed independent cross-verification, thereby maintaining methodological rigor in applying inclusion criteria. All retrieved studies received tripartite classification (‘include’; ‘exclude’; or ‘requiring further scrutiny’) through this collaborative process. Inter-rater discrepancies were addressed through structured deliberation during consensus-building sessions. For studies designated as requiring additional evaluation, the review team conducted iterative re-examinations of methodological details. When persistent interpretative variances occurred, arbitration by a senior third reviewer (with more than 5 years of systematic review experience) ensured conclusive resolution through evidence-based adjudication. A PRISMA flow chart detailing the selection process is presented in **Figure 1**.

### 2.5 Data extraction process

A pre-pilot extraction form was implemented using Microsoft Excel to systematically document study attributes and essential details from the reviewed literature. Key variables captured encompassed bibliographic information (author, publication year, country), study design characteristics (research objectives, methodological approach, participant demographics, outcome index), risk of bias indicators (e.g., randomization procedures, blinding status, attrition rates), and intervention effect.

The extraction framework underwent iterative refinement through a preliminary testing phase led by the primary investigator, ensuring standardization of data collection parameters across all included studies. To enhance methodological rigor, the principal researcher conducted initial data extraction, followed by a cross-verification process where co-authors independently validated the accuracy and completeness of recorded information through random sampling audits. This dual-phase approach maintained consistency in data capture while minimizing procedural bias.

Additionally, while formal quality appraisal is not mandated in scoping reviews, we conducted a targeted assessment of methodological rigor using the Downs & Black checklist (33) to provide readers with insights into the strengths and limitations of included studies. The checklist assesses study quality across five subscales: reporting, external validity, bias, confounding, and power. Raters answer specific questions, with scores reflecting the study’s methodological quality. The scoping review included all studies scoring above 18, a threshold established by the tool’s authors to indicate medium to high quality (see **Appendix 1**).

## 3 Results

### 3.1 Selection process

The systematic search initially yielded 14992 records, with 8014 remaining following duplicate removal. Title and abstract screening identified 2323 potentially eligible articles, of which 18 studies (20, 23, 34–49) met full inclusion criteria after rigorous full-text evaluation (**Figure 1**).

### 3.2 Study characteristics

The final corpus comprised 13 randomized controlled trials (20, 23, 34–37, 39–41, 43–45, 48), three quasi-experimental investigations (38, 46, 47), and two other studies (42, 49), collectively evaluating three intervention categories: cognitive-behavioral oriented interventions (n=5), emotion regulation and acceptance-oriented intervention (n=4), family-systemic oriented intervention (n=4), postmodern/constructivist-oriented intervention (n=1), resource-oriented and goal-focused intervention (n=1), pharmacological-behavioral combined intervention (n=2), and other interventions (n=1). Sample sizes ranged from 24 to 565 participants, with longitudinal follow-up durations spanning 1 month to 4 years (**Table 3**).

**Table 3:** Characteristics of included publications (n = 18).

(Author/Year) / Country	Study Design	Sample	Intervention Type	Intervention Details	Duration	Main Findings
Bjureberg et al. (42)/2018/Sweden	Uncontrolled open pilot trial	Study Start: N=60 Study End: N=50	Online ERITA	**IG:** Online ERITA (11 modules for adolescents + online parent program). No control group (TAU not specified).	12 weeks	Significant reductions in NSSI frequency (55% reduction,d= 0.88), NSSI versatility (d= 0.63), emotion dysregulation (d= 0.75), and improvements in global functioning.Effects maintained at 6-month follow-up. Mediation analysis confirmed emotion regulation as a key mechanism.
Livheim et al. (49)/2015/Sweden& Australia	Sweden: RCT; Australia: Planned comparison with random allocation for girls and one boys' group replication	Sweden: Study Start: N=32 (IG=15, CG=17); Study End: N=28 (IG=11, CG=14); Australia: Study Start: N=66 (IG=32 females + 8 males, CG=26 females); Study End: N=51 (IG=32, CG=19)	ACT-group intervention	Sweden (IG): 8-session ACT translated to Swedish, delivered after school; CG: Individual support by school nurses (2–5 sessions) Australia (IG): 8-session ACT manualized group program (experiential methods like painting/role-play); CG: 12-week monitoring support from school counselors	Sweden: 6 weeks (compressed schedule) Australia: 8 weeks	Sweden: Significant reduction in stress (large effect), marginally significant anxiety decrease and mindfulness improvement. Australia: Significant reduction in depressive symptoms (large effect) and psychological inflexibility (medium effect).
Hooley et al. (45)/2018/United States of America	RCT	Start: N=144 (ASET=49, EW=49, JNL=46); End: N=118 (ASET=39, EW=32, JNL=32)	Online Diary Intervention	IG=ASET (positive self-focus writing); CG1=EW (expressive writing); CG2=JNL (neutral journaling)	1 month	ASET reduced self-criticism post-treatment (not sustained); all groups showed reduced NSSI.
McCauley et al. (20)/2018/United States of America	RCT	Start: N=173 (DBT-A=86, IGST=87); End: N=118 (DBT-A=71, IGST=58)	Dialectical Behavior Therapy for Adolescents (DBT-A)	IG=DBT-A (individual+group therapy+family sessions); CG=IGST (supportive therapy)	6 months	DBT-A reduced suicide attempts and self-harm post-treatment, but group differences diminished at 12-month follow-up.
Dobias et al. (34) /2021/United States of America	RCT	Study Start: N=565 (IG286, CG=279); Study End: N=565 (IG=286, CG=279)	Online Single-Session Intervention (SSI)	IG: Project SAVE (CBT-based); CG: Supportive Therapy (Share Your Feelings SSI)	30 minutes	Project SAVE improved short-term self-hate and desire to stop NSSI but showed no long-term effects on self-injurious behaviors.
Lu et al. (43) China	RCT	Study Start: N=84 (IG=42, CG=42); Study End: N=80 (IG=40, CG=40)	Satir family therapy	**IG**: Sertraline (100 mg/d) + In-person and remote Satir family therapy **CG**: Sertraline (100 mg/d) + Routine health education	12 weeks	IG showed greater reductions in anxiety, depression, NSSI behaviors, and problematic phone use compared to CG.
Rockstroh et al. (35)/2023/ Switzerland& Germany	RCT	Study Start: N=74 (IG=37, CG=37); Study End: N=70 (IG=34, CG=36)	Cutting Down Programme (CDP)	**IG**: CDP (8–12 sessions of brief psychotherapeutic intervention) **CG**: enhanced TAU(non-manualized standard care)	8-12 sessions +2-4years follow-up	Both groups showed sustained NSSI reduction. IG demonstrated comparable long-term effectiveness to CG.
Asarnow et al. (37) /2017 /United States of America	RCT	Study Start: N=42 (IG=20, CG=22); Study End: N=42 (IG=20, CG=22)	Safety Program	**IG**: CBT + family therapy **CG**: enhanced TAU	12weeks +6-12 months follow-up	IG significantly reduced suicide attempts (NNT=3). No group differences in NSSI reduction.
Cheng et al. (46) /2024/China	Quasi-experimental study	Study Start: N=132 (IG=66, CG=66); Study End: N=118 (IG=61, CG=57)	Parent-child resilience training	**IG**: Group emotional regulation/resilience training + TAU **CG**: TAU	36 weeks	Both groups reduced NSSI frequency. IG showed greater improvements in hope, resilience, and family dynamics.
Wijana et al. (47) /2018/ Sweden	Quasi-experimental study	N=49 (IG=49, CG=N/A)	Intensive Contextual Therapy (ICT)	Integrated individual + family therapy	3months+6-12 months follow-up	Significant reductions in self-harm (d=0.54) and suicide attempts (d=1.38) post-intervention.
Liu et al. (48) /2025/ China	RCT	Study Start: N=100 (IG=50, CG=50); Study End: N=91 (IG=45, CG=46)	Sertraline + DBT-A vs. Sertraline + CBT	**IG**: Medication + dialectical behavior therapy **CG**: Medication + cognitive behavior therapy	12 weeks + 6 months	DBT-A group had lower NSSI frequency and better anxiety/depression outcomes at 6-month follow-up.
Zhang et al. (23) /2024/China	RCT	Study Start: N=60 (IG=30, CG=30); Study End: N=55 (IG=26, CG=29)	Narrative therapy	**IG**: Individual narrative therapy + routine care **CG**: routine psychological support	3 weeks + 1 month follow-up	IG showed greater reductions in NSSI frequency/severity and improved emotional outcomes compared to CG.
Chen et al. (40) /2023/ China	RCT	Study Start: N=93 (IG=46, CG=47); ​Study End: N=90 (IG=44, CG=46)	Phased behavioral intervention	**IG**: TTM-based staged intervention **CG**: routine care	6 months	IG showed significant improvement in depression, impulsivity, self-injury, and self-awareness vs. CG(*P*<0.05). Reduced NSSI behaviors and enhanced emotional regulation in IG.
Sinyor et al. (36) /2020/ Canada	Pilot RCT	Study Start: N=24 (IG=12, CG=12); Study End: N=21 (IG=11, CG=10)	Brief Cognitive Behavioral Therapy (BCBT) vs. Supportive Psychotherapy	**IG:** 10 acute sessions of BCBT (focused on suicide prevention, crisis response plans, emotion regulation, and relapse prevention) + 3 booster sessions. **CG:** Minimally directive supportive psychotherapy (non-specific emotional support).	15 weeks (acute phase) + 3 booster sessions+ 3-year follow-up.	BCBT significantly reduced repeat self-harm during acute follow-up (11% vs. 30% in CG, *P*<0.05). No between-group differences in suicidal ideation or depression scores at a 3-year follow-up.
Griffiths et al. (41) /2019/ The United Kingdom	RCT	Study Start: N=53 (IG=26, CG=27); Study End: N=48 (IG=22, CG=26)	Mentalization-Based Therapy (MBT-Ai) + TAU vs. TAU	**IG:** 12-week MBT-Ai group therapy (psychoeducation, self-reflection, role-play). **CG:** Routine care (psychotherapy, medication, case management).	12 weeks	Both groups reduced self-harm, anxiety, and borderline traits (*P*<0.05), but no between-group differences. Mentalization predicted reduced self-harm and hospital presentations (*P*<0.05).
Mehlum et al. (44) /2019/ Norway	A prospective 3-year follow-up of a randomized trial	Study Start: N=77(IG=39, CG=38) Study End: N=71 (IG=37, CG=34)	Dialectical Behavior Therapy for Adolescents (DBT-A) vs. Enhanced Usual Care (EUC)	**IG:** 19 weeks of DBT-A (skills training, family sessions, phone coaching). **CG:** EUC (weekly psychotherapy, pharmacotherapy).	19 weeks + 3-year follow-up	DBT-A showed sustained reduction in self-harm frequency vs. EUC at a 3-year follow-up (*P*<0.05). Hopelessness reduction mediated 70.8% of DBT-A’s effect on self-harm.
Chen et al. (38) /2024/ China	Quasi-experimental	Study Start: N=60 (IG=30, CG=30); Study End: N=60 (IG=30, CG=30)	Solution-Focused Brief Therapy (SFBT)	​**IG**: Routine care + SFBT (3 sessions over 2 weeks). **CG**: Routine care only.	2 weeks	Significant reduction in anxiety, depression, rumination, NSSI, and suicidal ideation scores in IG vs. CG (*P*<0.05). Higher scores in resource utilization and goal-oriented thinking (*P*<0.05)
Xu et al. (39) /2024/ China	RCT	Study Start: N=112 (IG=56, CG=56); Study End: N=112 (IG=56, CG=56)	rTMS + Sertraline	**IG:** High-frequency rTMS (10 Hz, 90% motor threshold, 10 min/day) +Sertraline (25-150mg/day). **CG:** sertraline (same dosage) + sham rTMS (coil placed perpendicularly to induce sound without cortical stimulation).	4 weeks	Significant reduction in HAMD-24 and SDS scores in IG vs. CG (*P*<0.05).IG showed lower self-injury frequency/severity and improved inflammatory markers (↓IL-1β, TNF-α; ↑IL-10) compared to CG.

Key: IG, Intervention Group; CG, Control Group; TAU, Treatment As Usual; NNT, Number Needed to Treat; TTM, Transtheoretical Model; rTMS, repetitive Transcranial Magnetic Stimulation; Online ERITA, Emotion Regulation Individual Therapy for Adolescents.

IG, Intervention Group; CG, Control Group.

#### 3.2.1 Intervention providers

Clinical interventions were predominantly delivered by multidisciplinary teams (n=8, 44%) integrating psychiatrists, clinical psychologists, nurses, and social workers (e.g., DBT-A, MBT-Ai, ICT). Licensed clinical psychologists independently delivered interventions in 28% of studies (n=5), while psychiatric physician-nurse teams accounted for 22% (n=4). A minority employed single-provider models (n=1, 6%) (e.g., narrative therapy).

#### 3.2.2 Intervention settings

Most interventions were hospital-based (n=10, 56%), primarily in outpatient clinics or inpatient wards. Community health centers supplemented delivery in 17% of studies (n=3), while 22% (n=4) utilized hybrid models combining in-person and teletherapy (e.g., remote Satir family therapy, web-based ERITA).

#### 3.2.3 Control group configurations

Comparator arms predominantly featured treatment-as-usual (TAU) protocols (n=10, 56%), encompassing standard pharmacotherapy, health education, or supportive therapy. Sham stimulation controls (e.g., rTMS placebo) and routine psychological support accounted for 11% (n=2) and 33% (n=6) of studies, respectively.

### 3.3 Classification and content of intervention strategies

Based on the core theoretical framework and operational mechanisms of interventions, this study classifies the interventions as follows:

#### 3.3.1 Cognitive-behavioral oriented interventions

Cognitive Behavioral Therapy (CBT) is a goal-oriented, systematic psychological intervention that integrates cognitive processes with behavioral strategies to help individuals modify maladaptive cognitive patterns and habitual behaviors, thereby alleviating emotional distress and mental health issues.

Short-term structured interventions grounded in CBT have gained widespread adoption due to their demonstrated efficacy and cost-effectiveness. The “Cutting Down Programme” (CDP), developed by Professor Rockstroh, employs a 10-session protocol that integrates crisis management planning with cognitive flexibility training. This intervention specifically targets maladaptive cognitive schemas and behavioral patterns associated with NSSI in adolescents, while simultaneously enhancing coping strategies and social support systems. The study found that both CDP and Treatment As Usual (TAU) showed comparable long-term effectiveness in reducing NSSI frequency (IRR=0.16), suicide attempts (IRR=0.27), and depressive symptoms (mean difference=-3.97), with no significant group differences observed (35).

Similarly, Brief Cognitive Behavioral Therapy (BCBT) (36) employs a structured protocol comprising 10 acute-phase sessions followed by 3 booster sessions. This intervention specifically targets the identification and modification of cognitive distortions and maladaptive behavioral patterns underlying self-injury, while systematically training patients in emotion regulation strategies and problem-solving skills. Empirical evidence from randomized controlled trials demonstrates that BCBT recipients exhibited significantly lower rates of recurrent self-injury during the acute treatment phase compared to control groups (OR=0.34, 95% CI [0.13–0.92]).

Chen implemented a stage-matched intervention guided by the Transtheoretical Model (TTM), deploying phase-specific strategies to address self-injurious behaviors (40). During the pre-contemplation phase, psychoeducational modules (e.g., video-based case studies illustrating long-term consequences of self-harm) were used to enhance risk perception. The contemplation phase incorporated motivational interviewing to resolve ambivalence by systematically weighing the pros/cons of behavioral change. In the ​preparation phase, clinicians co-developed individualized substitution behavior protocols (e.g., rubber band snapping to replace cutting) while addressing environmental triggers. The ​action/maintenance phase integrated family-monitored safety plans and ​mobile app prompts to reinforce adherence. This multi-tiered approach, combined with ​stage-contingent strategies (e.g., hazard restriction, family-involved contingency management), demonstrated efficacy in ​attenuating impulsivity and ​enhancing self-regulatory capacity through cognitive restructuring.

Moreover, the online single-session intervention (Project SAVE), targeting adolescents with recent self-injury, incorporates CBT principles through a 30-minute web-based program. It combines psychoeducation decoupling self-hatred from self-harm, peer testimonials on reduced self-injury, evidence-based alternative coping strategies (e.g., sensory substitution, cognitive reappraisal), and opportunities for participants to share personalized coping advice. While no significant effects on 3-month NSSI frequency were observed, short-term improvements in self-hatred (d=-0.35) and desire to stop self-harm (*d*=0.25) post-intervention surpassed control groups (34).

Additionally, Autobiographical Self-Enhancement Training (ASET) (45), a 28-day daily cognitive intervention, requires participants to document positive self-attributes (e.g., “I helped my neighbor today”) to reinforce self-worth and reduce self-criticism. Results show significantly lower self-criticism in ASET compared to neutral journaling (JNL) at treatment end (B=-4.31, *p*=0.047), with a trend toward reduced suicidal ideation at 3-month follow-up (B=-0.50, *p*=0.048).

#### 3.3.2 Emotion regulation and acceptance-oriented intervention

Dialectical Behavior Therapy for Adolescents (DBT-A) integrates individual and group-based therapeutic modalities to address emotion dysregulation and maladaptive coping behaviors. Individual therapy prioritizes crisis management by collaboratively developing personalized alternative self-soothing strategies (e.g., substituting self-harm with ice-cold stimulation for physiological grounding).

Concurrently, group therapy employs a structured four-step emotion regulation protocol—comprising ​emotion identification, ​nonjudgmental acceptance of present-moment experiences, adaptive behavioral selection, and reinforcement of positive outcomes—to cultivate metacognitive awareness. Role-playing simulations are systematically integrated to enhance interpersonal efficacy and conflict resolution skills, particularly in high-stakes social contexts. Two recent randomized controlled trials (20, 44) demonstrate DBT-A’s robust efficacy: intervention cohorts exhibited a 50% reduction in self-harm frequency compared to treatment-as-usual controls, with longitudinal follow-up (3-year) data revealing 50% lower relapse rates in self-injurious behaviors. Mechanistic analyses indicate these outcomes are mediated by ​enhanced emotion regulation capacity (e.g., reduced physiological hyperarousal during distress) and mitigated hopelessness through value-driven behavioral activation. This evidence underscores DBT-A’s dual focus on acceptance-based validation and skills-based behavioral change, positioning it as a first-line intervention for chronic emotion dysregulation and self-injurious phenotypes.

Acceptance and Commitment Therapy (ACT) (50)targets emotion dysregulation by fostering cognitive flexibility and values-driven behavioral commitment. Core interventions include: a) mindfulness practices to enhance present-moment emotional awareness; b) cognitive defusion techniques to reduce overidentification with negative emotions; and c) values-based behavioral activation. ACT utilizes cognitive defusion and values clarification to reduce adolescent avoidance of negative emotions and enhance engagement in value-driven behaviors. In an Australian quasi-randomized trial (N = 66; females randomized to ACT or control, males non-randomized), an 8-week ACT group intervention (using experiential methods like art and role-play) significantly reduced depressive symptoms and improved psychological flexibility (49).

In addition, the Emotion Regulation and Inclusion-Based Therapeutic Approach (ERITA) is a family-system intervention combining emotion identification training with bidirectional acceptance-oriented dialogue to disrupt intergenerational cycles of emotional suppression. Its framework includes: a) emotion diary exercises for precise labeling of complex emotions (e.g., “anger mixed with disappointment”), b) family workshops teaching nonjudgmental communication (e.g., replacing “You shouldn’t feel this way” with “I notice you’ve been tired lately” to reduce shame), and c) community partnerships (e.g., school-based stress-reduction groups). A pilot study (42) showed ERITA reduced Difficulties in Emotion Regulation Scale (DERS) scores by 42% versus controls, with mechanism analyses revealing its “bidirectional acceptance” model: Parents ceased suppressing adolescents’ emotions (e.g., “Don’t cry”) and actively reshaped dialogues (e.g., shifting “Why do you fail?” to “What did this setback make you feel?”). This dual transformation resulted in a 2.1-fold reduction in severe emotion regulation deficits (baseline DERS score>35; 95% CI:1.4–3.0; *p*<0.01), suggesting that family-systems approaches may be particularly effective for individuals with high-severity conditions.

#### 3.3.3 Family-systemic-oriented intervention

Family Function Training (46), involving parent-child group emotion regulation and resilience training (e.g., a 12-week modular course with gratitude, acceptance, and meaning-exploration units), positively affects adolescents’ hope, resilience, and family function. Strengthening parent-child bonds and building resilience in both adolescents and parents ​is a strategy that shows potential to reduce adolescents’ NSSI behavior.

Furthermore, Satir family therapy (43) utilizes role-playing and family sculpting to visualize emotional disconnections (e.g., simulating parental helplessness during self-injury), combined with non-accusatory communication protocols (e.g., reframing “you disappoint me” into “I need trust”) and crisis management strategies (e.g., collaborative “safe pause zones”). This approach not only reduces depressive/anxiety symptoms but also decreases comorbid smartphone dependency, highlighting multidimensional behavioral improvements.

Notably, the Safe Alternatives for Teens and Youths (SAFETY) program (37), a family-centered cognitive-behavioral intervention, represents the second psychosocial treatment (after I-CBT) with RCT-validated efficacy in reducing self-harm. Its framework integrates personalized safety planning, trigger identification, skill-building for adolescents and caregivers, and therapeutic consolidation, effectively enhancing family communication while lowering suicide risks. These findings underscore the multidimensional benefits of family-system approaches targeting both individual psychopathology and relational dynamics in NSSI management.

Grounded in ecological systems theory, Intensive Contextual Treatment (ICT) (47) integrates three intervention tiers:

- Individual: Weekly CBT sessions targeting emotional regulation and cognitive flexibility;
- Familial: Biweekly emotion-focused family therapy employing “I-statements” to replace accusatory communication;
- Social: School-based safety plans co-developed with counselors to mitigate environmental triggers (e.g., bullying response protocols).

The trial demonstrated a 46% reduction in non-suicidal self-injury frequency (*p*<0.001) with moderate effect size (*d*=0.71), alongside improved school adaptation correlating with reduced hospitalization days. Longitudinal data revealed symptom resurgence at 12-month follow-up, highlighting the necessity for enhanced continuity of care. ICT uniquely synthesizes principles from family therapy, dialectical behavior therapy (DBT-A), and CBT through structured modules addressing emotional dysregulation, familial communication, and social functioning. Its efficacy in reducing self-harm, suicide attempts, and internalizing symptoms remains supported across extended follow-up periods, underscoring its community applicability for adolescents with suicidality.

#### 3.3.4 Postmodern/constructivist-oriented intervention

Narrative therapy (NT), a postmodern psychotherapeutic approach, empowers adolescents with depression to deconstruct cultural oppression and rebuild self-identity by externalizing problems (e.g., reframing non-suicidal self-injury as “the pain controls you”) and reconstructing life narratives (23). Through “unique outcome” exploration, NT guides patients to recall successful non-self-injurious moments (e.g., “I managed stress without self-harm”) and reframe “exception events” (e.g., “I release emotions through exercise”), thereby enhancing self-efficacy and disrupting maladaptive identity-NSSI linkages.

#### 3.3.5 Resource-oriented and goal-focused intervention

In addition, Solution-Focused Brief Therapy (SFBT) is a therapeutic approach that facilitates individual change through structured conversations. Core techniques include: a) normalization techniques to depathologize distress, b) empowerment techniques to amplify self-efficacy beliefs, c) exception-seeking inquiry to identify preexisting adaptive behaviors, and d) miracle question exercises to visualize goal-directed futures. By redirecting patients’ focus toward intrinsic strengths and preexisting adaptive behaviors, SFBT facilitated co-constructing solution-oriented goals (e.g., “What small step aligns with your values today?”), which catalyzed sustained improvements in affective states and behavioral patterns (38).

#### 3.3.6 Pharmacological-behavioral combined intervention

A recent randomized trial (48) demonstrated the superior efficacy of sertraline combined with dialectical behavior therapy (DBT-A) versus cognitive-behavioral therapy (CBT) in reducing non-suicidal self-injury among adolescents with depression. The DBT-A cohort received weekly individual sessions, multifamily skills training, telephone coaching, and family therapy, while the CBT group underwent psychoeducation, emotion recognition, and behavioral activation. At 6-month follow-up, the DBT-A-sertraline group exhibited significantly greater reductions in NSSI frequency (42% vs. 28%, *p*<0.05) and comorbid anxiety/depressive symptoms compared to CBT-sertraline. This enhanced efficacy is attributed to DBT-A’s structured focus on emotion regulation and crisis management, synergizing with sertraline’s neurobiological effects to sustain long-term clinical improvements. The findings highlight the critical role of biological-behavioral synergy in optimizing NSSI treatment outcomes.

A study (39) demonstrated that high-frequency repetitive transcranial magnetic stimulation (HF-rTMS) combined with sertraline significantly reduced depressive symptoms and non-suicidal self-injury frequency in adolescents, concurrent with normalized inflammatory markers (↓IL-1β/TNF-α,↑IL-10; *p*<0.01). This dual intervention synergistically targets neuroinflammatory pathways and mood regulation circuits: HF-rTMS modulates dysfunctional neural circuitry while sertraline regulates serotonergic neurotransmission. The observed clinical improvements correlate with inflammatory rebalancing, suggesting a potential mechanistic link between cytokine modulation and behavioral outcomes. These findings highlight the therapeutic advantage of combined neuromodulatory and pharmacological approaches for NSSI-associated depression.

#### 3.3.7 Other interventions

Mentalization-Based Therapy for Adolescents (MBT-Ai) is a specialized group therapy adapted from the original MBT introductory manual. It focuses on enhancing adolescents’ mentalizing capacity by interpreting emotions, needs, and intentions through reflective dialogues (e.g., “How did you expect others to respond to your self-injury?”) and emotion-labeling exercises. By improving psychological understanding, it aims to replace self-harm with verbal expression. The intervention covers emotional literacy, mentalization principles, and attachment patterns.

MBT-Ai consists of 12 weekly 1.25-hour sessions. It was tested in a single-blind randomized trial involving adolescents with high interpersonal conflict. Results showed that the MBT-Ai group exhibited a 42% reduction in NSSI frequency and 60% fewer emergency visits compared to the control group at 12 weeks. Improved mentalization capacity was found to mediate therapeutic effects (*β*=0.32, *p*=0.02), underscoring its pivotal role in behavioral regulation (41).

### 3.4 Form and dosage of implementation

Implementation modalities predominantly employ in-person formats (**Table 4**), including individual psychotherapy, group sessions, family interventions, and neurostimulation techniques, with limited integration of telemedicine components (43). Self-monitoring strategies utilize structured diary-keeping to document daily emotional events and positive experiences, as seen in introspective and narrative-based approaches. Family-system interventions incorporate skill-building exercises such as emotion-focused communication drills, parent-adolescent dyadic regulation training, and collaborative problem-solving tasks.

**Table 4:** Summary table of implementation form and dosage.

Type of intervention	Site	Form of implementation	Frequency/Dose	Total course of treatment	Follow-up period
Online ERITA(Emotion Regulation Individual Therapy for Adolescents)	Online	Web platform + mobile app	Weekly modules	12 weeks	3- month and 6-month follow-ups
ACT-group intervention	high schools	Group sessions after school+ group sessions during school hours	Sweden: 8 sessions over 6 weeks, ~90 min/session; Australia: 8 sessions over 8 weeks, ~90 min/session	6-8 weeks	Not specified
Online Diary Intervention	Online	Daily writing tasks	5 min/day for 28 days	1 month	3 months
Dialectical Behavior Therapy (DBT-A)	Academic Medical Centers (4 sites)	Individual + Group Psychotherapy	Weekly sessions for 24 weeks	6 months	12 months
Online Single-Session Intervention (SSI)	Online(Nationwide USA)	Web-based program	Single session (30 minutes)	1 session	3 months
CDP (Brief Psychotherapy)	Outpatient	In-person + Individual	Average 10 sessions	Not specified	2–4 years
BCBT (Brief CBT)	Hospital	In-person + Individual	10 acute sessions + 3 booster sessions	15 weeks	6–12 months
TTM (Staged Intervention)	Inpatient	In-person + Phone follow-up + Staged modules	2×/week (inpatient), 3×/month (post-discharge)	6 months	6 months post-discharge
SFBT (Solution-Focused Brief Therapy)	Inpatient	In-person + Individual	Not specified	12 weeks	Immediate post-intervention
DBT-A (Dialectical Behavior Therapy for Adolescents)	Outpatient	In-person + Family + Homework	Weekly individual + Monthly family	12 months	3 years
Family Resilience Training	Outpatient	In-person + Family + Homework	Weekly	36 weeks	12/24/36 weeks
SAFETY (Family CBT)	Emergency/Inpatient	In-person + Family + Homework	Not specified	12 weeks	3 months
Satir Family Therapy	Hospital + Telehealth	In-person + Remote	Weekly	12 weeks	12 weeks post-intervention
MBT (Mentalization-Based Group Therapy)	Outpatient	In-person + Group	Weekly	12 weeks	6–12 months
rTMS + Sertraline	Inpatient	In-person + Individual	5×/week rTMS	4 weeks	6 months post-discharge
DBT-A + Sertraline	Hospital	In-person + Individual + Skills training	Weekly individual + Group	12 weeks	6 months
ICT (Integrated Family Therapy)	Outpatient	In-person + Skills training	Multiple weekly (unspecified)	12 weeks	12 months
Narrative Therapy (NT)	Inpatient	In-person + Individual + Self-monitoring	2×/week	3 weeks	1 month

Intervention duration varies by modality: Psychosocial protocols typically adopt 8–12-week frameworks with 1–2 weekly sessions (e.g., 12-session Mentalization-Based Therapy for Adolescents, 90 minutes/session). Pharmaco-behavioral combinations maintain comparable timelines (e.g., 12-week sertraline plus DBT-A) (48). Innovative models demonstrate compressed therapeutic cycles. This structured yet flexible implementation balances therapeutic intensity, familial engagement, and clinical pragmatism.

### 3.5 Outcome indicators and effects

The primary outcome measures were categorized into the following domains: clinical symptoms (including depression/anxiety, impulsivity, and related psychopathologies), self-injurious behaviors (encompassing frequency/severity and recurrence rates), psychosocial functioning (assessing self-perception and social adaptation), neurophysiological biomarkers (specifically neuroinflammation-associated markers such as TNF-*α*, IL-1β, and related cytokine profiles), along with other therapeutic-specific parameters (**Table 5**).

**Table 5:** Outcome indicators and effects of NSSI intervention strategies in adolescents with depression.

Intervention	Outcome category	Primary outcomes	Primary assessment tools	Reference
TTM (phased behavioral intervention)	1.Clinical symptoms	Depressive symptoms, impulsive behaviors	HAMD-24^1^, SDS^2^, BIS-II^3^;	Chen et al. (40)
	2.Self-injury behavior	Frequency and severity of self-harm	ASHQ^4^	
	3.self-cognition	Self-awareness level	PHSCS^5^	
High-frequency rTMS + Sertraline	1. Clinical Symptoms	Depression severity	HAMD-24^1^, SDS^2^	Xu et al. (39)
	2. Self-harm Behavior	Frequency of self-harm	ASHQ^4^	
	3.Physiological Indicators	Inflammatory cytokines (TNF-α, IL-1β, IL-10)	ELISA^6^	
Narrative Therapy	1. Self-harm Behavior	NSSI frequency and severity	CNRF^7^	Zhang et al. (23)
	2. Emotional Symptoms	Anxiety, depressive symptoms	SAS^8^, SDS^2^	
Solution-focused Model	1. Emotional Symptoms	Anxiety, depression, rumination	SAS^8^, SDS^2^, RRS^9^	Chen et al. (38)
	2. Self-harm-related Risks	Suicidal ideation, NSSI behaviors	PANSI^10^, NQ^11^	
	3. Cognitive Function	Solution-focused thinking (resource utilization, goal orientation)	SFI Scale^12^	
Dialectical Behavior Therapy (DBT-A)	1. Self-harm Behavior	Sustained reduction in self-harm episodes	LPC^13^	Mehlum et al. (44)
	2. Self-cognition	Hopelessness severity	BHS^14^	
	3. Clinical symptoms	Suicidal ideation	SIQ-JR^15^	
Sertraline + DBT	1. Self-injury behavior	NSSI frequency	DSHI-Y^16^	Liu et al. (48)
	2. Emotional Symptoms	Depression/anxiety symptoms	SDS², SAS⁸	
	3. Social Functioning	Personal/social performance	PSP^17^	
Family Therapy (Satir Model)	1. Clinical Symptoms	Anxiety and depression levels	SCARED^18^, DSRS^19^	Lu et al. (43)
	2. Behavioral Risks	NSSI behaviors, smartphone dependence	ANSSIAQ^20^, SQAPMPU^21^	
Parent-Child Group Intervention	1. Self-harm Behavior	NSSI frequency	OSI^22^	Cheng et al. (46)
	2. Psychological Resilience	Hope level, psychological resilience	HHS^23^, CD-RISC^24^	
	3. Family Functioning	Family adaptability, cohesion	FACES-II-CV^25^	
Cognitive Behavioral Therapy (CBT)	1. Self-harm Behavior	Suicidal ideation, self-harm recurrence	C-SSRS^26^, SSI^27^	Sinyor et al. (36)
	2. Clinical Symptoms	Depressive severity	MADRS^28^, BDI^29^	
	3. Social Functioning	Daily functional capacity	CIS^30^	
Mentalization-Based Therapy (MBT)	1. Self-harm Behavior	Frequency of self-harm	RTSHI^31^	Griffiths et al. (41)
	2. Emotional Regulation	Anxiety/depressive symptoms, reflective capacity	RCADS^32^, DERS^33^, RFQ-Y^34^	
Online ERITA (Emotion Regulation Individual Therapy for Adolescents)	1. Emotional Symptoms	Emotional management ability	DERS^33^	Bjureberg et al. (42)
	2. Self-injury Behavior	NSSI frequency and versatility	DSHI-9^35^	
	3. Family Functioning	Parental emotional validation	CCNES-A^36^	
	4. Social Functioning	Children’s global assessment	CGAS^37^	
Acceptance and Commitment Therapy (ACT)	1. Clinical symptoms	Depressive symptoms	RADS-2^38^	Livheim et al. (49), (Sweden Study)
	2. Self-Injury Behavior	Psychological inflexibility	AFQ-Y8^39^	
	3. Physiological Indicators	Stress levels	PSS^40^	
	4. Emotional Symptoms	Anxiety severity	DASS-S^41^	
	5. Psychological Resilience	Mindfulness skills	MAAS^42^	
ASET (Autobiographical Self-Enhancement Training)	1. Self-cognition	Self-criticism ability	SRS^43^	Hooley et al. (45)
	2. Self-injury behavior	NSSI frequency	Modified SITBI^44^	
	3. Clinical symptoms	Depression severity	BDI-II^45^	
Dialectical Behavior Therapy (DBT)	1. Self-injury behavior	Suicide attempts	SASII^46^	McCauley et al. (20)
	2. Clinical symptoms	Suicidal ideation	SIQ-JR^15^	
Project SAVE	1. Self-injury behavior	NSSI frequency	SITBI-R^47^	Dobias et al. (34)
	2. Self-cognition	Short-term reduction in self-hatred	SHS^48^	
Intensive Contextual Treatment (ICT)	1. Emotional Symptoms	Stress severity	YSR^49^	Wijana et al. (47)
	2. Self-harm Behavior	Self-harm frequency	DSHI-9R^50^	
	3. Social Functioning	School attendance	PSS^40^	
Cutting Down Programme (CDP)	1. Self-injury behavior	NSSI frequency	SITBI-G^51^	Rockstroh et al. (35)
	2. Clinical symptoms	Depression severity	BDI-II^45^	
SAFETY Program	1. Self-injury behavior	Self-harm frequency	C-SSRS^26^	Asarnow et al. (37)
	2. Family Functioning	Family support	Suicide History Interview	

^1^HAMD-24: 24-item Hamilton Depression Rating Scale.

^2^SDS: Self-Rating Depression Scale.

^3^BIS-II: Barratt Impulsiveness Scale, Version II.

^4^ASHQ: Adolescent Self-Harm Questionnaire.

^5^PHSCS: Piers-Harris Self-Concept Scale.

^6^ELISA: Enzyme-Linked Immunosorbent Assay.

^7^CNRF: Customized NSSI Recording Form.

^8^SAS: Self-Rating Anxiety Scale.

^9^RRS: Ruminative Response Scale.

^10^PANSI: Positive and Negative Suicide Ideation Inventory.

^11^NQ: NSSI Questionnaire.

^12^SFI Scale: Solution-Focused Inventory.

^13^HAMA: Hamilton Anxiety Rating Scale.

^14^BIS-11: Barratt Impulsiveness Scale-11.

^15^SHST: Self-Harm Screening Tool.

^16^SSPI Scale: Social Skills Performance Indicators.

^17^LPC: Lifetime Parasuicide Count.

^18^SIQ-JR: Suicide Ideation Questionnaire-Junior.

^19^MADRS: Montgomery-Åsberg Depression Rating Scale.

^20^DSHI-Y: Deliberate Self-Harm Inventory-Youth Version.

^21^CES-D: Center for Epidemiological Studies Depression Scale.

^22^MOAS: Modified Overt Aggression Scale.

^23^PSP: Personal and Social Performance Scale.

^24^SCARED: Screen for Child Anxiety Related Emotional Disorders.

^25^DSRS: Depression Self-Rating Scale.

^26^ANSSIAQ: Adolescent Non-Suicidal Self-Injury Assessment Questionnaire.

^27^SQAPMPU: Smartphone Application-Based Problematic Mobile Phone Use Questionnaire.

^28^OSI: Ottawa Self-Injury Inventory.

^29^HHS: Herth Hope Scale.

^30^CD-RISC: Connor-Davidson Resilience Scale.

^31^FACES-II-CV: Family Adaptability and Cohesion Evaluation Scale II, Chinese Version.

^32^C-SSRS: Columbia-Suicide Severity Rating Scale.

^33^SSI: Scale for Suicide Ideation.

^34^BDI-II: Beck Depression Inventory-II.

^35^CIS: Clinical Interview Schedule.

^36^RTSHI: Risk-Taking and Self-Harm Inventory.

^37^RCADS: Revised Child Anxiety and Depression Scale.

^38^DERS: Difficulties in Emotion Regulation Scale.

^39^RFQ-Y: Reflective Functioning Questionnaire for Youth.

^40^DSHI-9: Deliberate Self-Harm Inventory (9 items).

^41^CCNES-A: Coping with Children’s Negative Emotions Scale - Adolescent version.

^42^CGAS: Children’s Global Assessment Scale.

^43^RADS-2: Reynolds Adolescent Depression Scale-2.

^44^AFQ-Y8: Avoidance and Fusion Questionnaire-Youth (8 items).

^45^PSS: Perceived Stress Scale.

^46^DASS-S: Depression, Anxiety and Stress Scale.

^47^MAAS: Mindful Attention Awareness Scale.

^48^SRS: Self-Rating Scale.

^49^Modified SITBI: Modified Self-Injurious Thoughts and Behaviors Interview.

^50^SASII: Suicide Attempt Self-Injury Interview.

^51^SITBI-R: Self-Injurious Thoughts and Behaviors Interview-Revised.

^52^SHS: Self-Hate Scale.

## 4 Discussion

This scoping review systematically synthesizes evidence on interventions targeting adolescents with comorbid depression and non-suicidal self-injury (NSSI), addressing efficacy, contextual influences, and translational gaps. Our findings reveal three key themes aligned with the research questions (RQ).

### 4.1 Efficacy of interventions (RQ 1)

Dialectical Behavior Therapy for Adolescents (DBT-A) emerged as a cornerstone intervention, demonstrating sustained reductions in NSSI frequency (50% reduction vs. controls) and depressive symptoms through its dual focus on emotion regulation and crisis management (20, 44). Longitudinal trials highlight DBT-A’s capacity to reduce relapse rates by 50% over three years, mediated by enhanced physiological regulation (e.g., reduced hyperarousal) and mitigated hopelessness through value-driven behavioral activation (44, 48). However, the predominance of female participants in DBT-A trials (e.g., 94.8% female in McCauley et al., 2018) limits generalizability to males, who face higher suicide mortality rates—a critical limitation noted across studies.

Family-systemic interventions, such as Satir therapy and parent-child resilience training, improved family cohesion (FACES-II-CV scores: d=0.63) and reduced comorbid behaviors like smartphone dependency (43, 46). Pharmacological agents, particularly SSRIs, exhibited limited standalone efficacy for NSSI but demonstrated synergistic potential when combined with psychotherapy (48). For instance, neuromodulation-pharmacotherapy hybrids (e.g., rTMS + sertraline) achieved inflammatory cytokine normalization alongside behavioral improvements, implicating neuroimmune pathways in NSSI-depression comorbidity (39).

### 4.2 Contextual influences (RQ 2)

Cultural specificity remains a barrier: Studies like Lu et al. (43) were conducted in single-center Chinese hospitals, limiting applicability to Western or diverse contexts. Cross-cultural disparities in adherence (e.g., higher dropout rates in Western online interventions vs. Asian family therapies) highlight the need for localized protocols. Narrative therapy (23), reduced NSSI severity by externalizing self-injury as a “controllable adversary” (*d*=0.88). However, compressed intervention durations (e.g., 3-week protocols) and reliance on hospital-based settings (56% of studies) limit generalizability to community contexts.

### 4.3 Critical gaps (RQ 3)

Long-term follow-up data: Only 22% of studies tracked outcomes beyond 1 year, hindering insights into sustained efficacy. Digital health integration: Despite emerging evidence for telehealth (e.g., hybrid Satir therapy in Lu et al.) (43), only 17% of studies utilized digital platforms. The social contagion effects of online communities and the efficacy of app-based interventions (e.g., Project SAVE) (34) remain underexplored.

### 4.4 Strengths and limitations

#### 4.4.1 Strengths

##### 4.4.1.1 Methodological rigor

This review strictly adhered to the PRISMA-ScR guidelines, ensuring transparency in reporting and minimizing selection bias. Dual independent screening by four researchers (with cross-verification and arbitration by a senior reviewer) was implemented during study selection, enhancing reliability in applying inclusion criteria. Multi-database coverage (PubMed, PsycINFO, CNKI, Wanfang) and dual-language screening (Chinese/English) reduced regional bias and expanded evidence capture, particularly for non-Western interventions.

##### 4.4.1.2 Comprehensive scope

The inclusion of diverse intervention modalities (e.g., DBT-A, family therapy, pharmacotherapy) reflects real-world clinical complexity, aligning with the review’s aim to map heterogeneous evidence.

#### 4.4.2 Limitations

##### 4.4.2.1 Publication bias

The exclusion of grey literature (e.g., conference abstracts, unpublished trials) and non-English/Chinese studies may have omitted negative or region-specific findings, potentially skewing efficacy conclusions.

##### 4.4.2.2 Intervention heterogeneity

Variability in intervention designs (e.g., 3-week protocols vs. longitudinal family therapies) complicates direct comparisons. While this reflects clinical diversity, it underscores the need for replication studies to establish generalizable efficacy.

##### 4.4.2.3 Cultural and contextual gaps

Despite dual-language screening, 78% of family-system interventions were tested in East Asia (e.g., China), limiting insights into cultural adaptability for Western or low-resource settings. Notably, rural-urban disparities—particularly in mental health service accessibility—were not addressed in these trials.

### 4.5 Future directions

To address these gaps, we propose:

- Longitudinal, multi-center trials prioritizing male participants and culturally adapted protocols (e.g., DBT-A modules co-designed with local communities).
- Digital mental health frameworks integrating AI-driven chatbots for relapse prevention and blockchain-secured telehealth platforms.
- Interdisciplinary collaboration models (e.g., psychiatrist-educator partnerships) to bridge hospital-community divides.

## 5 Conclusion

This scoping review advances current understanding by systematically mapping interventions for adolescent depression with comorbid NSSI across diverse cultural and methodological contexts, addressing critical translational gaps. Psychotherapy, particularly dialectical behavior therapy for adolescents (DBT-A) and family-system approaches, demonstrates robust efficacy in reducing both depressive symptoms and NSSI behaviors. Novel findings include the synergistic potential of pharmacological-behavioral hybrids (e.g., rTMS + sertraline) in modulating neuroinflammatory pathways and the culturally adaptive benefits of family-system interventions in East Asian populations. However, real-world implementation readiness varies: DBT-A and digitally hybrid models (e.g., online ERITA) show immediate scalability in clinical settings, whereas compressed protocols (e.g., 3-week narrative therapy) require further validation. Critical gaps persist in long-term outcomes (only 22% of studies tracked more than 1-year effects), equitable digital health integration (only 17% utilized telehealth), and male-inclusive trials (94.8% female in DBT-A studies). To optimize precision frameworks, clinical priorities should prioritize DBT-A and family therapies; policy efforts must address digital divides and cultural adaptation; research agendas require longitudinal, community-based trials and AI-driven relapse prevention tools. Study limitations, including publication bias toward hospital-based interventions and underrepresentation of low-resource settings, underscore the need for systematic reviews to consolidate global evidence.
