# Supplementary material for: Interventions for adolescent depression comorbid with non-suicidal self-injury: a scoping review
Source: Front Psychiatry. 2025 Jun 26;16:1601073. doi: 10.3389/fpsyt.2025.1601073 (PMC12240963; doi:10.3389/fpsyt.2025.1601073)
Supplement: Supplementary file 1 [file DataSheet1.pdf]

Appendix 1 Critical appraisal of reviewed studies(n=18).

Quantitative Studies	Hooley et al. (2018)	Sinyor et al. (2020)	Rockstroh et al. (2023)	Dobias et al. (2021)	Chen et al. (2023)	Bjureberg et al. (2018)	Livheim et al. (2015)	Mehlum et al. (2019)	McCauley et al. (2018)	Asarnow et al. (2017)	Cheng et al. (2024)	Wijana et al. (2018)	Lu et al. (2023)	Zhang et al. (2024)	Chen et al. (2024)	Xu et al. (2024)	Liu et al. (2025)	Griffiths et al. (2019)
1: Is the hypothesis/aim/objective of the study clearly described?	Y	Y	Y	Y	Y	Y	Y	Y	Y	Y	Y	Y	Y	Y	Y	Y	Y	Y
2: Are the main outcomes to be measured clearly described in the Introduction or Methods section?	Y	Y	Y	Y	Y	Y	Y	Y	Y	Y	Y	Y	Y	Y	Y	Y	Y	Y
3: Are the characteristics of the patients included in the study clearly described ?	Y	Y	Y	Y	Y	Y	Y	Y	Y	Y	Y	Y	Y	Y	Y	Y	Y	Y
4: Are the interventions of interest clearly described?	Y	Y	Y	Y	Y	Y	Y	Y	Y	Y	Y	Y	Y	Y	Y	Y	Y	Y
5: Are the distributions of principal confounders in each group of subjects to be compared clearly described?	Y	P	Y	Y	P	N	P	Y	Y	Y	N	Y	Y	Y	P	Y	Y	Y
6: Are the main findings of the study clearly described?	Y	Y	Y	Y	Y	Y	Y	Y	Y	Y	Y	Y	Y	Y	Y	Y	Y	Y
7: Does the study provide estimates of the random variability in the data for the main outcomes?	Y	Y	Y	Y	Y	Y	Y	Y	Y	Y	Y	Y	Y	Y	Y	Y	Y	Y
8: Have all important adverse events that may be a consequence of the intervention been reported?	N	N	N	N	N	Y	N	N	Y	N	N	N	N	N	N	N	N	Y
9: Have the characteristics of patients lost to follow-up been described?	N	N	N	N	N	Y	Y	Y	Y	Y	Y	Y	N	Y	N	N	Y	Y
10:Have actual probability values been reported(e.g. 0.035 rather than <0.05) for the main outcomes except where the probability value is less than 0.001?	Y	Y	Y	Y	Y	Y	Y	Y	Y	Y	N	Y	N	Y	Y	N	Y	Y
11. Were the subjects asked to participate in the study representative of the entire population from which they were recruited?	UTD	UTD	UTD	UTD	UTD	UTD	UTD	UTD	UTD	UTD	UTD	UTD	UTD	UTD	UTD	Y	UTD	UTD
12: Were those subjects who were prepared to participate	UTD	UTD	UTD	UTD	UTD	UTD	UTD	UTD	UTD	UTD	UTD	UTD	UTD	UTD	UTD	UTD	UTD	UTD

representative of the entire population from which they were recruited?																		
13: Were the staff, places, and facilities where the patients were treated, representative of the treatment the majority of patients receive?	Y	N	Y	N	N	Y	Y	N	N	Y	Y	N	Y	N	N	UTD	UTD	Y
14: Was an attempt made to blind study subjects to the intervention they have received?	N	N	N	N	N	N	N	N	N	N	N	N	Y	N	N	Y	N	N
15: Was an attempt made to blind those measuring the main outcomes of the intervention?	UTD	Y	Y	Y	UTD	UTD	N	Y	Y	Y	Y	UTD	UTD	N	N	UTD	UTD	Y
16：If any of the results of the study were based on “data dredging”, was this made clear?	Y	Y	Y	Y	Y	Y	Y	Y	Y	Y	Y	Y	Y	Y	Y	Y	Y	Y
17：In trials and cohort studies, do the analyses adjust for different lengths of follow-up of patients, or in case-control studies, is the time period between the intervention and outcome the same for cases and controls?	Y	Y	Y	Y	Y	Y	Y	Y	Y	Y	Y	Y	Y	Y	Y	Y	Y	Y
18: Were the statistical tests used to assess the main outcomes appropriate?	Y	Y	Y	Y	Y	Y	Y	Y	Y	Y	Y	Y	Y	Y	Y	Y	Y	Y
19: Was compliance with the intervention/s reliable?	Y	Y	Y	Y	Y	Y	Y	Y	Y	Y	Y	Y	Y	Y	Y	Y	Y	Y
20: Were the main outcome measures used accurate (valid and reliable)?	Y	Y	Y	Y	Y	Y	Y	Y	Y	Y	Y	Y	Y	Y	Y	Y	Y	Y
21: Were the patients in different intervention groups (trials and cohort studies) or were the cases and controls (case-control studies) recruited from the same population?	Y	Y	Y	Y	Y	UTD	Y	Y	Y	Y	Y	Y	Y	Y	Y	Y	Y	Y
22: Were study subjects in different intervention groups (trials and cohort studies) or	Y	Y	Y	Y	Y	Y	UTD	Y	Y	Y	Y	Y	UTD	Y	Y	Y	Y	Y

were the cases and controls (case-control studies) recruited over the same period of time?																		
23: Were study subjects randomised to intervention groups?	Y	Y	Y	Y	Y	N	Y	Y	Y	Y	Y	N	Y	Y	N	Y	Y	Y
24: Was the randomised intervention assignment concealed from both patients and health care staff until recruitment was complete and irrevocable?	UTD	N	UTD	Y	UTD	N	N	N	Y	N	N	N	Y	N	N	N	N	Y
25: Was there adequate adjustment for confounding in the analyses from which the main findings were drawn?	Y	Y	Y	Y	N	N	Y	Y	Y	Y	Y	N	Y	Y	N	Y	Y	Y
26: Were losses of patients to follow-up taken into account?	Y	Y	Y	Y	Y	Y	Y	Y	Y	Y	Y	Y	UTD	Y	UTD	UTD	Y	Y
27. Did the study have suffcient power to detect a clinically important effect where the probability value for a difference being due to chance is less than 5%?	2	1	1	3	2	2	2	1	3	0	4	2	5	2	3	5	2	1
Score	22	20	22	24	19	19	21	22	26	22	23	20	23	22	19	24	22	25

Q1- Q4& Q6-26: YES (1) = Y; NO (0) = N; Unable to determine (0) =UTD.

Q5: YES (1) = Y; PARTIAL (1) = P.

Q27: (Size of smallest intervention group $<n_1$) (0)=A; (Size of smallest intervention group: n_1-n_2) (1)=B; (Size of smallest intervention group: n_3-n_4) (2)=C ; (Size of smallest intervention group: n_5-n_6) (3)=D; (Size of smallest intervention group: n_7-n_8) (4)=E; (Size of smallest intervention group: n_8+) (5)=F.
